# Supplementary material for: Rapid and Sensitive Detection of Toxigenic Fusarium asiaticum Integrating Recombinase Polymerase Amplification, CRISPR/Cas12a, and Lateral Flow Techniques
Source: Int J Mol Sci. 2023 Sep 15;24(18):14134. doi: 10.3390/ijms241814134 (PMC10531391; doi:10.3390/ijms241814134)
Supplement: Supplementary file 1 [file ijms-24-14134-s001.zip › Table S1.pdf]

Table S1 Primers and guide RNA used in this study.

Name	Sequence (5’-3’)	Length (bp)
RPA- CYP51C-F	TTATGTCAAAACCTCGCCATACTTCAAGGGCG	31
RPA- CYP51C-R	TCCATTACCTTGCGGGCGTGGTTCCGACGA	30
RPA-CYP51C -crRNA	UAAUUUCUACUAAGUGUAGAUCUAGGAGGGACC CUGAUGCUG	42
ssDNA reporter	5’-FAM-TTATT-biotin-3’	5
