# Supplementary material for: Rapid and Sensitive Detection of Toxigenic Fusarium asiaticum Integrating Recombinase Polymerase Amplification, CRISPR/Cas12a, and Lateral Flow Techniques
Source: Int J Mol Sci. 2023 Sep 15;24(18):14134. doi: 10.3390/ijms241814134 (PMC10531391; doi:10.3390/ijms241814134)
Supplement: Supplementary file 1 [file ijms-24-14134-s001.zip › Table S2.pdf]

Table S2 Fungal strains used in this study and their results in the RPA-Cas12a-LFD assay.

Species	Host	Location in China	RPA-Cas12a-LFD
***Fusarium* strains**			
Fusarium temperatum	Maize	Yunnan, Liaoning	+
Fusarium boothii	Maize	Yunnan, Inner Mongolia, Shanxi	-
Fusarium oxysporum	Maize	Liaoning	-
Fusarium verticillioides	Maize	Jilin, Inner Mongolia, Shandong, Liaoning, Shanxi , Shanghai, Gansu, Henan	-
Fusarium graminearum	Maize	Jilin, Yunnan	-
Fusarium cortaderiae	Maize	Yunnan	-
Fusarium proliferatum	Maize	Liaoning, Henan	-
Fusarium fujikuroi	Maize	Henan	-
Fusarium andiyazi	Maize	Inner Mongolia	-
Fusarium avenaceum	Maize	Yunnan	-
Fusarium meridionale	Maize	Yunnan	-
Fusarium Solani	Maize	Yunnan	-
Fusarium subglutinans	Maize	Inner Mongolia, Shanxi	-
Fusarium equiseti	Maize	Inner Mongolia	
***Non-Fusarium* strains**			
Aspergillus niger	Maize	Inner Mongolia	-
Aspergillus costaricensis	Maize	Gansu	-
Alternaria spp.	Maize	Inner Mongolia	-
